# The Mental Health Risks of Adolescent Cannabis Use

**DOI:** 10.1371/journal.pmed.0030039

**Published:** 2006-01-24

**Authors:** Wayne Hall

## Abstract

Wayne Hall summarizes the research evidence on whether cannabis use is associated with psychiatric problems.

Since the early 1970s, when cannabis first began to be widely used [ 1], the proportion of young people who have used cannabis has steeply increased and the age of first use has declined [ 2, 3]. Most cannabis users now start in the mid-to-late teens [ 1], an important period of psychosocial transition when misadventures can have large adverse effects on a young person's life chances.

Dependence is an underappreciated risk of cannabis use [ 1]. There has been an increase in the numbers of adults requesting help to stop using cannabis in many developed countries, including Australia [ 4] and the Netherlands [ 1, 5]. Regular cannabis users develop tolerance to many of the effects of delta-9-tetrahydrocannabinol [ 6–8], and those seeking help to stop often report withdrawal symptoms [ 9–11]. Withdrawal symptoms have been reported by 80% of male and 60% of female adolescents seeking treatment for cannabis dependence [ 12, 13].

In epidemiological studies in the early 1980s [ 14] and 1990s [ 15], it was found that 4% of the United States population had met diagnostic criteria for cannabis abuse or dependence at some time in their lives. Surveys in Australia, Canada, and New Zealand have produced similar estimates [ 16–19]. About one in ten of those who use cannabis meet criteria for dependence [ 15], but this risk is much higher for daily users and persons who start using at an early age [ 20, 21]. Only a minority of cannabis-dependent people in surveys report seeking treatment (Chapter 7 of [ 1]), but among those who do, fewer than half succeed in remaining abstinent for as long as a year [ 9, 10, 22].

Those who use cannabis more often than weekly in adolescence are more likely to develop dependence, use other illicit drugs, and develop psychotic symptoms and psychosis [ 1]. Establishing whether cannabis use is a contributory cause of these outcomes [ 1] requires two things: (1) longitudinal research on the effects that cannabis use in adolescence has on psychosocial outcomes in young adulthood [ 23], and (2) statistical methods to control for the fact that young people who regularly use cannabis differ from their peers who do not in ways that increase regular cannabis users' risk of these adverse psychosocial outcomes [ 1, 23].

## Is Cannabis a Gateway Drug?

Surveys of adolescents in the United States over the past 30 years have consistently shown [ 24] that: (1) almost all adolescents who had tried cocaine and heroin had first used alcohol, tobacco, and cannabis, in that order [ 25, 26], (2) regular cannabis users are the most likely to use heroin and cocaine [ 27], and (3) the earlier the age of first cannabis use, the more likely a young person is to use other illicit drugs [ 26].

Three explanations have been offered for these patterns: that those who use cannabis at an early age are more likely for other reasons to use other illicit drugs; that cannabis users obtain the drug from the same black market as other illicit drugs (providing more opportunities to use these drugs); and that the effects of cannabis on the brain increase an adolescent's propensity to use other illicit drugs [ 28].

Animal studies of the neurobiology of drug effects provide some biological plausibility for a causal relationship in showing that cannabis and other illicit drugs act on the same brain reward centres [ 29–31]. Nevertheless, the role that cannabis plays remains controversial because it is difficult to exclude the hypothesis that these patterns of use are substantially explained by the personal characteristics of cannabis users and the shared environments that make those who use cannabis more likely to use other drugs [ 24, 32].

Several studies have recently suggested that the pattern cannot be wholly explained in this way. A well-controlled longitudinal study of a birth cohort found that the pattern did not disappear after statistical adjustment for plausible common factors [ 33]. Two studies of twins who were discordant for cannabis use have found that the relationship between cannabis use and the use of other illicit drugs persisted after controlling for the effects of shared genes and environment [ 34, 35].

## Cannabis Use and Psychosis

Cannabis use and psychotic symptoms and disorders are associated in the population [ 37–38] and in persons with schizophrenia [ 39–41]. The major explanations of this association have been that: (1) cannabis use precipitates schizophrenia in persons who are vulnerable to the disorder [ 1], (2) cannabis is used to self-medicate symptoms of schizophrenia, or (3) the association arises from uncontrolled confounding by variables that predict an increased risk of both cannabis use and schizophrenia [ 23, 42].

The first explanation was supported by a 15-year prospective study of 50,465 Swedish conscripts [ 43] that found a dose–response relationship between the risk of developing schizophrenia and the number of times cannabis had been used by age 18. These risks remained significant after statistical adjustment for confounding variables. A later 27-year follow-up of this cohort [ 44] also found a dose–response relationship between the frequency of cannabis use and the risk of schizophrenia, which persisted after statistically controlling for confounding factors.

These findings have been recently replicated in: a three-year study of 4,848 young people in the Netherlands [ 45]; a 4-year follow up of a cohort of 2,437 young Germans [ 46]; and two New Zealand birth cohorts ( *n* = 759 [ 47]; *n* = 900 [ 48]). All of these studies found a relationship between regular cannabis use and psychosis (with a RR of 2–3) that persisted after controlling for confounding variables [ 49].

In the Dutch, German and New Zealand cohorts, young people who reported psychotic symptoms at baseline were much more likely to report psychotic symptoms at follow-up if they used cannabis than were cannabis-using peers who did not report these symptoms at baseline. In one of the New Zealand studies, young people with a variant allele of the COMT gene who used cannabis had a risk of reporting psychotic symptoms that was ten times higher than young people who did not have the allele who used cannabis [ 50].

The self-medication hypothesis was not supported in the van Os or Henquet studies [ 45, 46], both of which found that early psychotic symptoms did not predict an increased use of cannabis. These results have been supported by Verdoux et al. [ 51], who found that cannabis users were more likely to report unusual perceptions after using cannabis than to report using cannabis in response to experiencing unusual perceptions, and that this relationship was stronger in individuals with a history of psychotic experiences.

## Communicating the Risks

In most developed countries, the debate about cannabis policy is often simplified to a choice between two options: (1) to legalize cannabis because its use is harmless, or (2) to continue to prohibit its use because it is harmful [ 1]. As a consequence, evidence that cannabis use causes harm to adolescents is embraced by supporters of cannabis prohibition and is dismissed as “flawed” by proponents of cannabis liberalization (e.g., [ 52]).

A major challenge in providing credible health education to young people about the risks of cannabis use is in presenting the information in a persuasive way that accurately reflects the remaining uncertainties about these risks. The question of how best to provide this information to young people requires research on their views about these issues and the type of information they find most persuasive. It is clear from US experience that it is worth trying to change adolescent views about the health risks of cannabis; a sustained decline in cannabis use during the 1980s was preceded by increases in the perceived risks of cannabis use among young people [ 53].

The following are brief summaries of the evidence intended for health professionals.

### Cannabis dependence

Cannabis users can become dependent on cannabis. The risk (around 10%) is lower than that for alcohol, nicotine, and opiates, but the earlier the age a young person begins to use cannabis, the higher the risk.

### Cannabis and other illicit drug use

Regular users of cannabis are more likely to use heroin, cocaine, or other drugs, but the reasons for this remain unclear. Some of the relationship is attributable to the fact that young people who become regular cannabis users are more likely to use other illicit drugs for other reasons, and that they are in social environments that provide more opportunities to use these drugs. It is also possible that regular cannabis use produces changes in brain function that make the use of other drugs more attractive. The most likely explanation of the association between cannabis and the use of other illicit drugs probably involves a combination of these factors.

### Cannabis and psychosis

As a rule of thumb, adolescents who use cannabis more than weekly probably increase their risk of experiencing psychotic symptoms and developing psychosis if they are vulnerable—if they have a family member with a psychosis or other mental disorder, or have already had unusual psychological experiences after using cannabis. This vulnerability may prove to be genetically mediated.

## 

**Figure:**
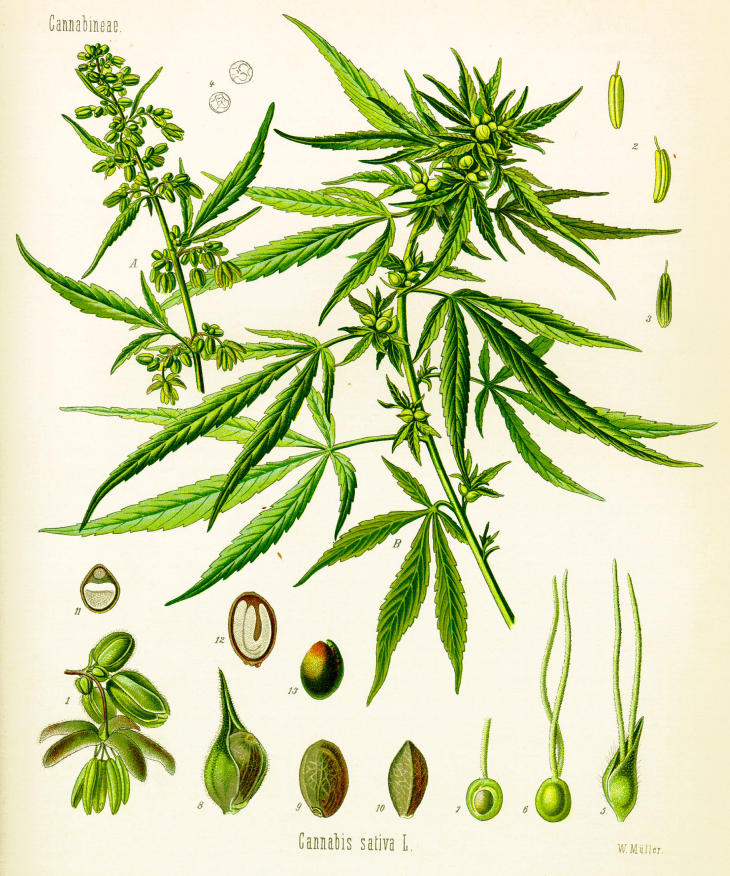
Scientific drawing of *Cannabis sativa*, circa 1900
